# Estimating prevalence from dried blood spots without using biological cut-offs: application of a novel approach to hepatitis C virus in drug users in France (ANRS-Coquelicot survey)

**DOI:** 10.1017/S0950268819001043

**Published:** 2019-06-17

**Authors:** L. Léon, J. Pillonel, M. Jauffret-Roustide, F. Barin, Y. Le Strat

**Affiliations:** 1 Santé publique France, French national public health agency, F-94415 Saint-Maurice, France; 2 Cermes3 (Inserm U988/CNRS UMR8211/EHESS/Université Paris Descartes), Paris, France; 3 UMR Inserm U1259, Université de Tours, & CNR VIH, CHU Bretonneau, Tours, France

**Keywords:** Cut-off, drug users, hepatitis C virus, mixture model, prevalence

## Abstract

Seroprevalence estimation using cross-sectional serosurveys can be challenging due to inadequate or unknown biological cut-off limits of detection. In recent years, diagnostic assay cut-offs, fixed assay cut-offs and more flexible approaches as mixture modelling have been proposed to classify biological quantitative measurements into a positive or negative status. Our objective was to estimate the prevalence of anti-HCV antibodies among drug users (DU) in France in 2011 using a biological test performed on dried blood spots (DBS) collected during a cross-sectional serosurvey. However, in 2011, we did not have a cut-off value for DBS. We could not use the values for serum or plasma, knowing that the DBS value was not necessarily the same. Accordingly, we used a method which consisted of applying a two-component mixture model with age-dependent mixing proportions using penalised splines. The component densities were assumed to be log-normally distributed and were estimated in a Bayesian framework. Anti-HCV prevalence among DU was estimated at 43.3% in France and increased with age. Our method allowed us to provide estimates of age-dependent prevalence using DBS without having a specified biological cut-off value.

## Introduction

In epidemiology, accurately estimating indicators such as prevalence is crucial to study and monitor a disease in the general public and specific populations, and to implement appropriate public health measures. One approach to estimate the prevalence of a specific infection is to ask people to self-report their infection as part of a survey [1]. However, only people aware of their infection, who fully understand the question and who agree to disclose their status, can do so. Therefore, the most suitable way to estimate prevalence is to conduct an epidemiological survey in which a biological assay specific to this infection is performed on all survey participants. Such assays are most often performed by laboratories on biological samples (or matrices) from well-defined populations (such as blood donors). The assay cut-off value – established using reference material and standard conditions (biological matrices (traditionally, serum or plasma), commercial biological kits and storage) – is often provided by the manufacturer. This cut-off value allows the classification of biological quantitative measurements into a ‘positive’ result (infection or past infection), a ‘negative’ result (not infected) or an ‘inconclusive’ result. In the latter, a reanalysis of the biological sample is necessary, otherwise the sample is considered positive or negative or excluded from the epidemiological study, or remains inconclusive.

According to this classification, estimating seroprevalence is usually straightforward if the assay is performed under the conditions recommended by the manufacturer [2].

The sensitivity and the specificity of a biological assay can be modified if not performed according to the manufacturer's instructions. In an epidemiological survey, the choice of the format of biological/serological assay to use depends on several factors. The most important of these are: target population, ease of use, implementation conditions of the survey (whether within dedicated care/prevention services or outside of such services), testing laboratory and cost. Seroprevalence estimates may be biased when a biological assay is applied to a population and/or to a biological matrix different from those used to calibrate and validate the assay [2–5].

To avoid inconclusive classifications arising from the use of fixed or arbitrary biological cut-off values, an approach using a mixture of distributions – to model the distribution of the biological quantitative measurements – was proposed in the literature [6]. Called the ‘direct approach’, it differs from the classic cut-off approach [7].

In this article, our objective was to estimate the prevalence of anti-HCV antibodies (anti-HCV) among drug users (DU) in France, from a biological test performed on dried blood spots (DBS) collected during the ANRS-Coquelicot survey in 2011 [8, 9]. However, in 2011, we did not have a cut-off value for DBS and we could not use the values for serum or plasma, knowing that the DBS value was not necessarily the same. The distribution of the biological quantitative measurements (signal-to-cut-off (s/co) ratios) used to detect anti-HCV antibodies was estimated using a Bayesian framework incorporating a two-component mixture model with age-dependent mixing proportions using penalised splines [10]. We compared our results with three other approaches (two model-based approaches published recently [8] and the classic biological cut-off approach).

## Methods

### Data sources

ANRS-Coquelicot was a French cross-sectional serosurvey performed in 2011 among DU recruited in five French metropolitan cities (Lille, Strasbourg, Paris, Bordeaux and Marseille) and two administrative departments in the Paris area (Seine-Saint-Denis and Seine-et-Marne) [9]. Inclusion criteria were as follows: individuals >18 years who had injected or snorted drugs ‘at least once in his/her life’, spoke French and agreed to participate in the survey (providing informed consent). The survey's main objectives were to estimate the prevalence of anti-HIV and anti-HCV antibodies, to assess at-risk practices associated with HCV transmission and to evaluate the dynamics of the HIV and HCV epidemics in this population. DU were selected using time-location sampling [11] and interviewed about their socio-demographic situation, health status, access to HIV and HCV screening, knowledge of HIV and HCV transmission modes, drug use in their lifetime and in the previous month, at-risk practices and access to care. In the present sub-study, we focus on the estimation of anti-HCV prevalence. Blood samples on blotting paper were collected during the interview by participants who agreed to provide self-obtained finger-prick blood samples on DBS for anti-HIV and anti-HCV antibody testing.

### Biological samples

Screening for anti-HCV antibodies was performed using ELISA with the HCV 3.0 Ortho assay (Raritan, NJ, USA). Details on the serological data analysis are available in a previous paper [8]. Briefly, the DBS were cut out with a punch to obtain a circle 6 mm in diameter, which was first placed in 250 µl of 0.01 M sodium phosphate buffer containing 10% bovine serum albumin and 0.05% Tween 20, then incubated at room temperature for 1 h in an ultrasonic cleaner. The eluted serum samples were directly used to fill the wells of ELISA microplates (200 µl per well). Subsequent steps were carried out in strict compliance with the manufacturer's recommendations. We used the s/co ratios (i.e. absorbance over the manufacturer's cut-off) to measure the concentration of anti-HCV antibodies. For quantitative analysis, absorbance, also known as optical density (a measure of the quantity of light absorbed by a sample), was measured using spectroscopy. Manufacturers establish cut-off values from absorbance readings of negative (and sometimes positive) controls. The s/co ratio enables serological samples to be normalised, as slight variations may arise from one set of manipulations to another. Manufacturers usually define samples with an s/co ratio of ≥ 1.0 as positive.

### Mixture modelling

For each DU *i* (*i* = 1, …, *n*), let us consider his age *a*_*i*_ and the variable of interest *y*_*i*_ corresponding to the log-transformed concentration of anti-HCV antibodies. We modelled the variable of interest using a two-component mixture model, each component representing seropositive or seronegative DU for HCV. Following the methodology and the notations used by Vink *et al*. [10], each DU *i* contributes to the likelihood: *f* (*y*_*i*_) = (1 − *I*_*i*_) *f*_0_ (*y*_*i*_) + *I*_*i*_*f*_1_ (*y*_*i*_) with *I*_*i*_ equals zero if *i* belongs to the seronegative component *f*_0_ and *I*_*i*_ equals one if *i* belongs to the seropositive component *f*_1_. We assumed that the random variable *I*_*i*_ followed a Bernoulli distribution of parameter *p*_*i*_ where *p*_*i*_ was the age-dependent probability of being seropositive. To estimate *p*_*i*_, we performed a logit regression including age modelled by penalised splines:
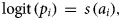
where the smooth function of age *s* is a *B*-penalised-spline model. The term *s* (*a*) can be expressed by: *s* (*a*) = *BXβ* + *BZb*. Here *B* is a *n* × *k* cubic-*B*-spline with *k* equally spaced knots, *X* is a *d* × *k* matrix such as *X* *β* is a polynomial of degree *d* − 1 and *Z* = *D*^*T*^ (*DD*^*T*^)^−1^ is a*k* × (*k* − *d*) matrix, where *D* is a (*k* − *d*) × *k* difference matrix of order *d*. *β* is a vector of length *d* and *b* is a vector of length *k* − *d*. In our study, we put knots on 10 equally distributed age groups and penalised second-order differences (i.e. *k* = *10* and *d* = *2*).

Each component density *f*_*j*_ was assumed to be normally distributed, independent of age, with mean *μ*_*j*_ and standard deviation *σ*_*j*_ where *j* = 0 (resp. *j* = 1) was associated with the seronegative component (resp. seropositive component).

We used weakly-informative priors for the unknown parameters. Parameters were estimated using the Bayesian framework through Gibbs sampling, using Just another Gibbs Sampler (JAGS) [12]. We ran four parallel Markov chain Monte Carlo (MCMC) models and retained 5000 samples in total. Details of the estimation procedure are available elsewhere [10] and our R code is provided in the Appendix.

### Mixture model validation

To assess whether the model provided a reasonable fit of the data by age group (18–25, 26–35, 36–45 and 46–55), first, we calculated the empirical cumulative distribution function (ECDF) of the data with the 95% confidence intervals. The procedure to generate the confidence intervals consists of using an asymptotic approximation (Approximate Critical Values for Kolmogorov–Smirnov's D) [13]. Second, we plotted the ECDF, the 95% CI and the predicted ECDF obtained from the posterior predictive distribution of the data according to the log concentration of anti-HCV antibodies. For the latter model, we used the estimated parameters 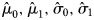 and the estimated seroprevalence per age group, calculated as the weighted mean of the age-specific seroprevalences.

### Estimation of the prevalence

To produce estimates in the DU population, all the analyses took into account the sampling design (sampling weights, stratifications, primary sampling units).

**Fig. 1.**
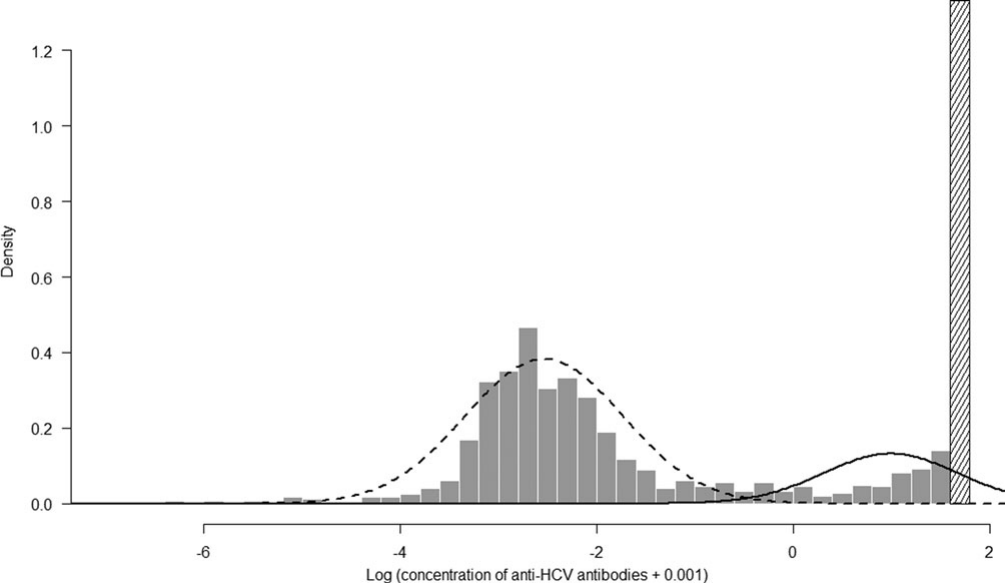
Results of the 2-component mixture model performed on the log transformed-concentration (log(x+0.001)) without the results over the upper limit of absorbance of the spectrophotometer. The dashed curve represents the component for the seronegative results and the solid curve represents the component for the seropositive results. The solid bars represent the distribution of quantitative results and the last hashed bar represents the results over the upper limit of absorbance of the spectrophotometer (≥ 10.0).

A biological result was indicated ‘over’ when the quantitative measurement exceeded the upper limit of absorbance of the spectrophotometer (≥10.0). It is a very frequent phenomenon with any ELISA. The signal reaches quickly a plateau when a sample is strongly positive. There is a continuous antigenic stimulation by HCV antigens in any chronically infected individual that leads to a high level of antibody. This is why most of the positive samples are ‘over’ the plateau. ‘Over’ results were excluded from the mixture model for two reasons: (1) they did not need to be classified because they were obviously defined as positive; (2) keeping them would involve an artificial distribution of the quantitative results with right censored data impacting the mixture model (Fig. 1). We defined *n*_over_ as the number of DU with an anti-HCV concentration result indicated ‘over’. Furthermore, anti-HCV prevalence was estimated in two steps: (1) estimation of prevalence, 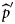 using the mixture model excluding the ‘over’ results, and then (2) estimation of the final prevalence 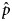 including the ‘over’ results.

#### Step 1. Estimation of prevalence 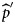 using the mixture model excluding the ‘over’ results

We estimated the probability of being seropositive at age *a*, 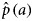, by the mean probability of being seropositive for age *a* from the 5000 generated samples. Using 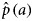 and the estimated proportion of DU by age, we calculated the prevalence 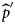. The proportion of DU of age *a*, denoted by *q* (*a*), was estimated using the Horvitz–Thompson estimator 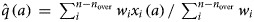, where *w*_*i*_ is the sampling weight of the individual *i*, *x*_*i*_ (*a*) = 1 if the individual *i* is of age *a* and 0 otherwise, and where *n* is the survey sample size [14]. The prevalence was expressed by 
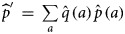
.

#### Step 2. Estimation of the final prevalence 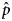


The final estimated prevalence 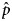 was obtained with the formula: 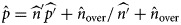.


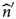
 is the estimated number of DU with a biological quantitative result and 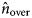 is the estimated number of DU with a concentration result indicated ‘over’.

### Comparison of different approaches proposed to estimate the prevalence

Four methods were compared:

- *Model 1*: estimation using the two-component Bayesian mixture model presented above,
- *Model 2*: estimation using a five-component EM mixture model. Each component corresponded to a level reactivity. Levels 1–3, corresponding to the lowest reactivity, represented the negative results of the anti-HCV test. Levels 4–5 were assumed to represent the positive results of the test [8],
- *Model 3*: estimation using a logistic regression model as a function of age and time [8],
- *Model 4*: estimation using the classic biological cut-off method, using a cut-off value of one. Indeed, there was no cut-off value for DBS assay but the cut-off value for serum was equal to 1. We used this cut-off value assuming that the cut-off value was the same on DBS or serum which is a strong assumption.

All analyses were performed using R 3.3.2 software and the running time to perform computations was about 3 min using a standard personal computer.

## Results

In the ANRS-Coquelicot survey, 1568 DU were included and 92% of the respondents agreed to provide a finger-prick blood sample, corresponding to 1442 blotting papers collected. Two hundred blotting papers were excluded due to insufficient or inadequate biological material. A total of 1242 DBS samples were retained for the analysis [8]. Among them, 289 DU had a concentration result indicated ‘over’, and were thus classified anti-HCV seropositive.

### Mixture model and validation model

**Fig. 2.**
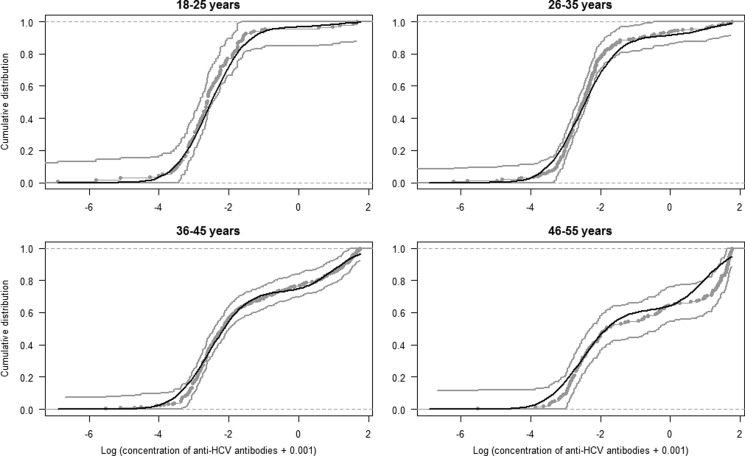
Model fit. Curves represent the empirical cumulative distribution function and 95% CI of the anti-HCV log-transformed concentration per age group (gray curves) and the model predicted cumulative distribution function (black curve).

The estimated parameters of the mixture model are shown in Table 1 and the normal density functions of the components (*f*_0_ *and* *f*_1_) in Figure 1. Figure 2 shows, for each age group, the ECDF of the anti-HCV log concentration with the model-predicted cumulative distribution function. For each age group, the model-predicted result stands between the 95% confidence intervals of the empirical results.

**Table 1.** Parameters of the mixture model

Component		Status	*μ*_*i*_	Credible intervals	*σ*_*i*_	Credible intervals
*f*_0_		Seronegative	−2.54	−2.64 to −2.46	0.8	0.75–0.87
*f*_1_		Seropositive	0.99	0.53–1.15	0.69	0.56–0.84

### Estimation of the prevalence

**Fig. 3.**
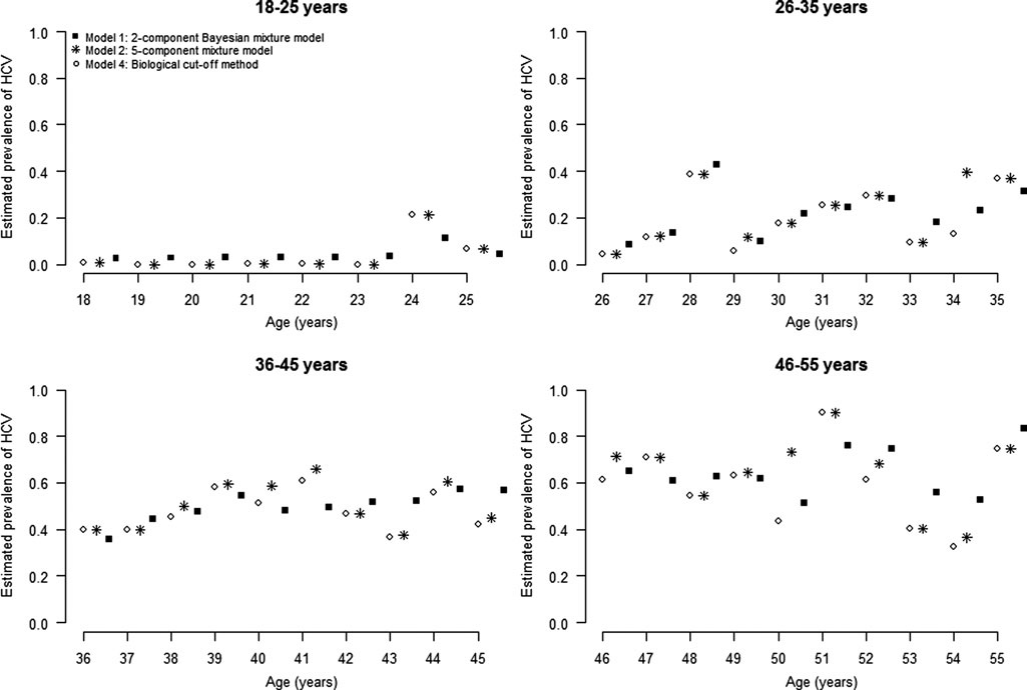
Age-dependent HCV prevalence estimates, from the 5-component mixture model (stars), from the 2-component Bayesian mixture model (squares) and using the biological cutoff method (circles).

**Fig. 4.**
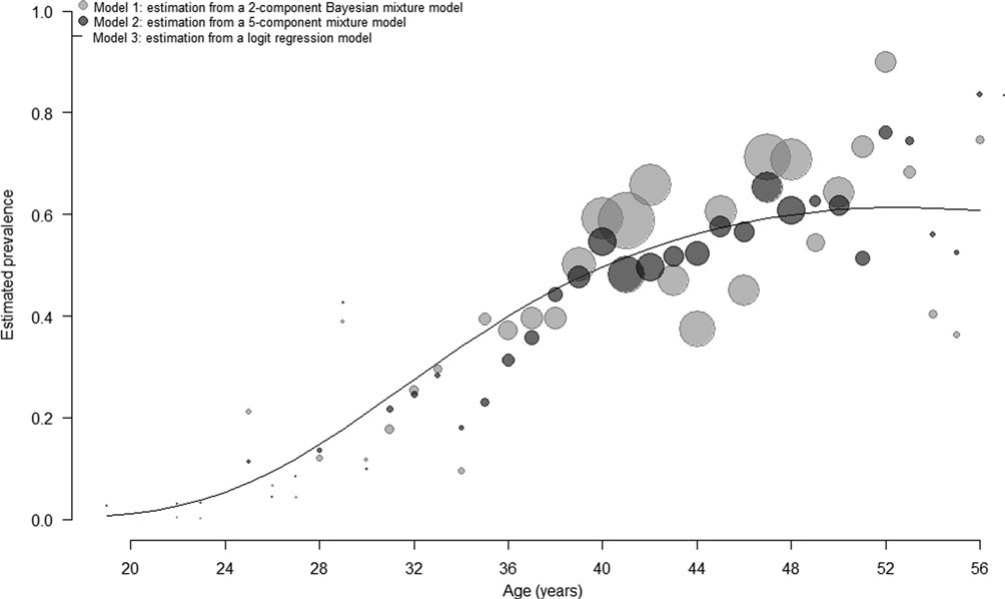
Age-dependent HCV prevalence estimates, from a 5-component mixture model (light gray circles), from a logit regression model (line) and from the 2-component Bayesian mixture method (dark gray circles). The circles’ sizes are proportional to the number of participants.

We estimated anti-HCV prevalence among DU in France at 43.3% (95% CI 36.2–50.3). As expected, anti-HCV prevalence increased with age (Fig. 3). We compared our current method to estimate age-dependent prevalence of HCV with three other methods: (1) estimation using a five-component mixture model [8], (2) estimation using a logistic regression model [8] and (3) estimation using the biological cut-off approach (Table 2 and Figs 3 and 4). In the first of these three methods (*model 2*), each of the five components corresponded to an anti-HCV reactivity level. We observed similar results between our current method and two other methods: *model 2* and *model 3*. Using a cut-off value of 1.0, the biological cut-off approach provided a lower HCV prevalence, estimated at 39.9% (95% CI 35.9–44.1). Most estimates by age group were lower using the third method (*model 4*) than using our reference method (Table 2).

**Table 2.** Comparison of four different approaches to estimate HCV prevalence among drug users, ANRS-Coquelicot survey, France, 2011

Age group	Model 1		Model 2		Model 3		Model 4	
	Two-component mixture	Credible intervals	Five-component mixture	95% CI	logit regression	95% CI	Biological cut-off	95% CI
Total	43.3	36.2–50.3	43.2	38.8–47.7	43.5	42.1–44.9	39.9	35.8–44.1
18–25	5.4	1.4–9.3	6.3	2.2–16.6	5.5	5.0–6.1	6.3	2.2–16.9
26–35	24.3	16.9–25.7	21.8	15.9–29.0	27.0	26.0–28.4	19.0	13.6–25.7
36–45	49.5	44.8–54.2	50.6	44.5–56.7	51.6	50.9–52.3	47.9	41.7–54.1
46–55	63.6	59.2–68.1	67.5	58.3–75.4	60.6	60.5–60.8	60.8	52.8–68.2

## Discussion

In serosurveys, biological assays are used to estimate seroprevalence. The choice of specific biological assays and the practical conditions have an impact on the estimation of seroprevalence. Usually, they depend on diagnostic cut-off values, biological matrices used and the studied populations. Traditionally, virological assays are assessed using serum or plasma samples collected from venous puncture. The advent of alternative biological matrices to venous blood samples, such as gingival crevicular fluid (taken from the lip and gum), capillary blood (taken from the finger), hair samples and home urine specimens, means that biological testing can now occur in the patient's home environment (point of care testing) [15, 16]. DBS samples are often preferred for collecting biological measurements in hard to reach or vulnerable populations at high risk of infectious diseases [17]. For example, venipuncture is inadequate for epidemiological studies on homeless people [18], men who have sex with men [19] and injecting DU, due to venous access sometimes being difficult because of repeated injections [20] and to study implementation protocols (e.g. data collection for DU performed by outreach teams in the streets). Cut-off values using these alternative biological matrices are usually different from those for sera samples. They are not easy to obtain and sometimes are not provided by the biologist [2, 3, 5, 17, 21]. If the cut-off value is unknown, the serological status will be subject to misclassification due to an arbitrary choice of the cut-off value. An inaccurate use of the manufacturer's instructions can lead to a different sensitivity and specificity than those expected. This has a direct impact on positive/negative classification, and in turn on the estimation of seroprevalence [21]. When two cut-off values (upper and lower) are provided by the manufacturer, individuals with a result under the lower cut-off value are considered seronegative, those with a result over the upper cut-off value seropositive and those with a result between the two inconclusive [22].

In our point of view, the estimation of prevalence using biological measurements can be treated statistically without using a cut-off value, and therefore can be viewed as a statistical challenge rather than a biological one.

Today, mixture models are used for various diseases to determine the cut-off value for diagnostic reasons [23], to estimate seroprevalences [10, 24, 25] and to differentiate different levels of antibody reactivity [24, 26]. This article presents a method for estimating anti-HCV prevalence by age, without using an assay cut-off, an arbitrary cut-off or a level reactivity of anti-HCV detection. We used an age-dependent normal mixture model on the log-transformed anti-HCV concentrations, without classifying measurements as positive or negative. Parameters were estimated using a Bayesian framework. We estimated anti-HCV prevalence among DU in France at 43.3% (95% CI 36.2–50.3). This work follows various attempts to estimate the prevalence of hepatitis C among DU in France.

In 2011, because DBS had lower sensitivity to detect anti-HCV antibodies and because of inconsistency in some classifications (i.e. where some DU testing negative (i.e. s/co ratio ≤ 1.0) were classified anti-HCV antibody seropositive or HCV cured, because their s/co ratio was close to 1.0), the DBS cut-off value was modified. Specifically, a group was formed composed of biologists, epidemiologists, sociologists and biostatisticians to propose a DBS cut-off value. This group proposed a classification algorithm based on DU characteristics and s/co ratio values. The recommendation was to choose a threshold value for DBS samples at 0.64 IU/l. Using this empirical cut-off value, Weill-Barillet *et al*. estimated the HCV prevalence at 44% (95% CI 39.6–47.9), which was very close to our estimate [9].

This first approach having shown that the choice of the biological cut-off could be difficult, we tried other approaches.

The naïve approach (corresponding to model 4) was to choose a cut-off value equal to 1 (as used for serum), knowing that it was a highly improbable value.

We then tried to estimate the prevalence from a logistic regression including age and time as covariates (model 3). However, the variable of interest was the result of a binary classification of the biological quantitative measurements, which still did not allow to estimate a prevalence by avoiding the choice of a cut-off value.

We then applied a five-component mixture model (model 2), using the standard EM algorithm for normal mixtures, which maximises the conditionally expected complete-data log-likelihood at each M-step of the algorithm [8]. HCV prevalence among DU using that model (model 2) was estimated at 43.2% (95% CI 38.8–47.7), close to our HCV prevalence estimate (model 1) [8]. The main limitation of this five-component model is to have to choose, a posteriori, which components should be considered as corresponding to the negative or the positive HCV status. This choice is quite arbitrary and can be influenced by the results obtained with the previous approaches.

The approach proposed in this paper overcomes this difficulty. Unlike the five-component mixture model and the logit regression model, our model does not need a classification step (i.e. classifying positive or negative status) to estimate anti-HCV antibody seroprevalence. We do not have to choose a threshold to treat posterior probabilities.

However, fitting a mixture model to quantitative measurements may be difficult for a number of reasons: the possibility that a low proportion of measurements may belong to one of the underlying components, the possibility of a visually unimodal distribution of quantitative measurements, the difficulty to choose normal distributions *vs.* other distributions (such as the skew-normal) or the interest to take into account biological characteristics of individuals (vaccination, co-infection). For this last point, these characteristics can be included as covariables in a multivariate regression model to explain the probabilities of being seropositive.

Despite limitations specific to the use of mixture modelling, our approach remains a valid alternative method to estimate prevalence from serosurvey data when one has no information on assay cut-off value. Our method can be applied to populations with special characteristics (e.g. at high risk of infectious diseases, co-infected individuals and children) or with a serological response different to the population(s) used to calibrate biological tests. Furthermore, our approach allows a detection cut-off value to be estimated, when the traditional conditions for using biological tests are modified or unavailable (blood samples, laboratory conditions, study populations, etc.) and may contribute to help biologists, public health researchers and decision-makers.

## Supplementary material

For supplementary material accompanying this paper visit https://doi.org/10.1017/S0950268819001043. click here to view supplementary material
