# Supplementary material for: Estimating prevalence from dried blood spots without using biological cut-offs: application of a novel approach to hepatitis C virus in drug users in France (ANRS-Coquelicot survey)
Source: Epidemiol Infect. 2019 Jun 17;147:e220. doi: 10.1017/S0950268819001043 (PMC6625185; doi:10.1017/S0950268819001043)
Supplement: Supplementary file 1 [file S0950268819001043sup001.docx]

**APPENDIX**

**R code for estimating the mixture model parameters using JAGS**

# Set working directory

setwd("<path>")

#--------------------------

# Load packages

#--------------------------

library(splines)

library(parallel)

library(rjags)

#--------------------------

# Setup cluster

#--------------------------

n.cores <- detectCores()

cl <- makeCluster(n.cores) # makeCluster creates a set of copies of R running in parallel and communicating over sockets.

#--------------------------

# Functions

#--------------------------

**# JAGS function for parallel computing (Just Another Gibbs Sampler)**

# 1/ define the model using the BUGS language in a separate file: see model.string below

jags.par <- function(X, data, inits, variable.names, n.iter, n.burnin, thin) {

library(rjags)

# 2/ read the model using the jags.model function

jags.mod <-jags.model(file = "model.txt", data = data,

inits = inits, n.chains = 1, n.adapt = 500, quiet = TRUE)

# 3/ update the model using the update function. This creates an object "jags".

update(jags.mod, n.iter = n.burnin)

#4/ extract samples form the model object using the coda.samples function. This creates an object "mcmc.list".

coda.samples(model = jags.mod, variable.names = variable.names,n.iter = n.iter, thin = thin)

}

**# Mixed model P-spline setup function**

PSplineSetup <- function(x, x.min = min(x), x.max = max(x),k = 10, spline.deg = 3, diff.ord = 2) {

# B-spline basis

dx <- (x.max - x.min)/(k-spline.deg)

knots <- seq(x.min - spline.deg*dx, x.max+spline.deg*dx, by = dx)

B <- spline.des(knots = knots, x = x, ord = spline.deg+1, outer.ok = TRUE)$design # new function now: SplineDesign

# Difference operator matrix

D <- diff(diag(k), diff = diff.ord) # diag(k) construit une matrice diagonale à k lignes avec des 1 sur la diagonale

# Re-parameterize B and D into X (fixed effects) and Z (random effects)

X <- B%*%outer(knots[1:k], 0:(diff.ord - 1), "^")

Z <- B%*%t(D)%*%solve(tcrossprod(D))

return(list(X = X, Z = Z, n = nrow(X), q = ncol(X), m = ncol(Z)))

}

**# Calculation of the 95%CI of the ECDF for the log concentration of antiHCV antibodies**

cuminc <- function(x, mu0, mu1, sd0, sd1, w){

(1-w)*pnorm(x, mean = mu0, sd = sd0) + w*pnorm(x, mean = mu1, sd = sd1)

}

ecdf.ksCI.adj <- function (x, main = NULL, sub = NULL, xlab = deparse(substitute(x)),

ci.col = "red", ...)

{

force(xlab)

stopifnot(require(stats))

if (is.null(main))

main <- paste("ecdf(", deparse(substitute(x)), ") + 95% K.S. bands",

sep = "")

n <- length(x)

if (is.null(sub))

# sub <- paste("n = ", n)

ec <- ecdf(x)

xx <- get("x", envir = environment(ec))

yy <- get("y", envir = environment(ec))

D <- KSd(n)

yyu <- pmin(yy + D, 1)

yyl <- pmax(yy - D, 0)

ecu <- stepfun(xx, c(yyu, 1))

ecl <- stepfun(xx, c(yyl, yyl[n]))

plot(ec, main = main, sub = sub, xlab = xlab , xlim = c(resume["Min."],resume["Max."]), col = ci.col)

plot(ecu, add = TRUE, verticals = TRUE, do.points = FALSE,

col.hor = ci.col, col.vert = ci.col, lwd = 2, ...)

plot(ecl, add = TRUE, verticals = TRUE, do.points = FALSE,

col.hor = ci.col, col.vert = ci.col, lwd = 2, ...)

}

#--------------------------

# Data

#--------------------------

# import the data

dataset

# data modification

dataset$loghcv <-log(dataset$hcv +0.001)

# infectious status: to be estimated

dataset$inf<-NA

#--------------------------

# P-spline setup

#--------------------------

# P-spline setup

x <- dataset

psp.data <- PSplineSetup(x = x$age-37, x.min = 18-37, x.max = 65-37, k = 10)

# Predictions

psp.pred <- PSplineSetup(x = 18:65-37, x.min = 18-37, x.max = 65-37, k = 10)

#--------------------------

# Fit mixture model with JAGS

#--------------------------

# Model

model.string <- "model {

# Likelihood data

for (i in 1:n.P2) {

y.P2[i] ~ dnorm(mu[inf.P2[i]+1], tau[inf.P2[i]+1])

inf.P2[i] ~ dbern(p.P2[i])

logit(p.P2[i]) <- sum(X.P2[i, 1:q]*beta.P2[1:q]) + sum(Z.P2[i, 1:m]*b.P2[1:m])

}

# Predictions

for (i in 1:n.pred) {

logit(p.pred.P2[i]) <- sum(X.pred[i, 1:q]*beta.P2[1:q]) + sum(Z.pred[i, 1:m]*b.P2[1:m])

}

# Prior random effects

for (j in 1:m) {

b.P2[j] ~ dnorm(0, tau.b.P2)

}

# Hyperprior random effects

tau.b.P2 ~ dgamma(1, 0.01)

# Prior fixed effects

for (j in 1:q) {

beta.P2[j] ~ dnorm(0, 0.01)

}

# Prior Normal components

mu[1] ~ dnorm(0, 0.01)

mu[2] <- mu[1]+d.mu

d.mu ~ dnorm(0, 0.01)I(0, )

tau[1] ~ dgamma(1, 0.01)

tau[2] ~ dgamma(1, 0.01)

}"

# Data

data.list <- list(

n.P2 = psp.data$n,

q = psp.data$q,

m = psp.data$m,

y.P2 = dataset$loghcv,

inf.P2 = dataset$inf,

X.P2 = psp.data$X,

Z.P2 = psp.data$Z,

n.pred = psp.pred$n,

X.pred = psp.pred$X,

Z.pred = psp.pred$Z)

# Inits

inits.fun <- function() with(data.list, list(

b.P2 = rep(0, m),

tau.b.P2 = 1,

beta.P2 = rep(0, q),

mu = c(0, NA),

d.mu = 3,

tau = rep(1, 2),

.RNG.name = "base::Wichmann-Hill", .RNG.seed = sample(1:10000, 1) # RNG = Random Number Generator

))

#--------------------------

# Run JAGS

#--------------------------

# The function ‘mcmc.list’ is used to represent parallel runs of the same chain,

# with different starting values and random seeds. The list must be balanced:

# each chain in the list must have the same iterations and the same variables.

writeLines(model.string, "model.txt")

jags.model(file = "model.txt", data = data.list, inits = inits.fun, n.chains = 1, n.adapt = 500)

clusterExport(cl, varlist = "data.list")

post.mcmc <- as.mcmc.list(parSapply(cl, X = 1:4, FUN = jags.par, data = data.list,

inits = inits.fun, n.iter = ceiling(50000/4), n.burnin = 500, thin = 10, # 50000/4=12500

variable.names = c(

"tau.b.P2",

"beta.P2",

"mu",

"tau",

"p.pred.P2",

"deviance", "

b.P1","inf.P2"

)

))
